# Simvastatin Does Not Affect Vitamin D Status, but Low Vitamin D Levels Are Associated with Dyslipidemia: Results from a Randomised, Controlled Trial

**DOI:** 10.1155/2010/957174

**Published:** 2009-07-21

**Authors:** Lars Rejnmark, Peter Vestergaard, Lene Heickendorff, Leif Mosekilde

**Affiliations:** ^1^Department of Endocrinology and Metabolism C, Aarhus Sygehus, Aarhus University Hospital, 8000 Aarhus, Denmark; ^2^Department of Clinical Biochemistry, Aarhus Sygehus, Aarhus University Hospital, 8000 Aarhus, Denmark

## Abstract

*Objectives*. Statin drugs act as inhibitors of the 3-hydroxy-3methylglutaryl coenzyme A (HMG-CoA) reductase enzyme early in the mevalonate pathway, thereby reducing the endogenous cholesterol synthesis. In recent studies, it has been suggested from epidemiological data that statins also may improve vitamin D status, as measured by increased plasma 25-hydroxyvitamin D (25OHD) levels. We now report the results from a randomised controlled trial on effects of simvastatin on plasma 25OHD levels. *Design and Methods*. We randomised 82 healthy postmenopausal women to one year of treatment with either simvastatin 40 mg/d or placebo and performed measurement at baseline and after 26 and 52 weeks of treatment. The study was completed by 77 subjects. *Results*. Compared with placebo, plasma levels of cholesterol and low-density lipoproteins decreased in response to treatment with simvastatin, but our study showed no effect of simvastatin on vitamin D status. However, plasma levels of triglycerides were inversely associated with tertiles of plasma 25OHD levels and changes in plasma triglycerides levels correlated inversely with seasonal changes in vitamin D status. *Conclusion*. Our data do not support a pharmacological effect of statins on vitamin D status, but do suggest that vitamin D may influence plasma lipid profile and thus be of importance to cardiovascular health.

## 1. Introduction

During recent years, treatment with statins has been suggested to cause positive effects on bone. Although, discrepant results have been reported in clinical studies on effects of statins on bone mineral density (BMD) and bone turnover [1–5], several epidemiological studies have shown that treatment with statins is associated with a reduced risk of fracture [6–10]. Several mechanisms of action of statins on bone have been suggested. Statins may exert a bone anabolic action due to an increased osteoblastic synthesis of bone morphogenetic protein 2 (BMP-2), a growth factor that causes osteoblastic proliferation [11], as well as antiresorptive effects similar to nitrogen-containing bisphosphonates (amino-BP) [12]. Moreover, an effect of statins on vitamin D metabolism has been suggested as an additional mechanism of action by which statins may exert pleiotropic effects. In several [13–16] but not all [17–19] studies, treatment with statins has been associated with an improved vitamin D status. An emerging amount of evidence suggests that an impaired vitamin D status increases the risk of different types of cancers and chronic disorders, including cardiovascular diseases [20, 21]. If statins improve vitamin D status this could be a plausible explanation for the findings of not only a decreased fracture risk, but also a decreased risk of malignant diseases in users of statin drugs [22]. Thus, in a randomised controlled design we studied effects of one year of simvastatin treatment on vitamin D status in a group of healthy postmenopausal women.

## 2. Subjects and Methods

In year 2000, we initiated a study on effects of statin treatment on bone. The design and major results of the study has previously been detailed [1]. In brief, in a double-blinded design we randomised 82 healthy Caucasian women to one year of treatment with either simvastatin 40 mg/d or placebo. In addition, all studied subjects received a daily supplement with 400 mg of elementary calcium, but no vitamin D supplementations. Calcium and simvastatin tablets were obtained commercially.

We recruited studied subjects, through invitations by letter, from a random sample of the general background population. We only included women who were more than 12 months postmenopause and below 76 years of age. In addition, we required studied subjects to be healthy as assessed by a standard biochemical screening program and to have osteopenia at the lumbar spine or total hip, that is, a BMD less than 1 standard deviation (SD) below the mean of peak bone mass (T-score < −1). We excluded women with diseases or use of drugs known to affect calcium homeostasis and bone metabolism, including impaired renal (plasma creatinine >120 *μ*mol/L) or hepatic (plasma alanine aminotransferase >80 U/L) function, and alcohol abuse of more than 14 units a week within the last 2 years. None of studied subjects had known hyperlipidemia prior to study start. As a safety measure, we excluded subjects with low plasma cholesterol levels (total cholesterol <4.0 mmol/L and/or LDL <2.5 mmol/L).

The study was carried out in accordance with the Declaration of Helsinki II. It was approved by the regional Ethical Committee (Aarhus County # 2000/0223) and the Danish National Board of Health. Each individual gave verbal and written informed consent prior to the study. The Good Clinical Practice (GCP) Unit at the University Hospital of Aarhus, Denmark, monitored the study.

## 3. Biochemistry

During trial, blood samples were drawn between 7.00 a.m. and 10.30 a.m. after an overnight fast. At time of study, we analysed plasma levels of calcium, creatinine, albumin, total cholesterol (TC), LDL cholesterol (LDL), HDL cholesterol (HDL), and triglyceride (TG) by standard laboratory methods. We now report measurements of plasma 25-hydroxyvitamin D (P-25OHD) levels in samples collected at baseline and after 6 and 12 months of treatment. Since collected, all samples have been stored at −80°C. We analysed P-25OHD levels using an isotope dilution liquid chromatography-tandem mass spectrometry (LC-MS/MS) method adapted from Maunsell et al. [23]. Mean coefficients of variation for 25OHD3 were 6.4% and 9.1% at levels of 66.5 and 21.1 nmol/L and for 25OHD2 the CV values were 8.8% and 9.4% at levels of 41.2 and 25.3 nmol/L.

## 4. Statistics

We assessed differences between study groups using Chi-square tests for categorical variables and a two-sample *t*-test or Mann-Whitney *U*-test for continuous variables, as appropriate. For this analysis, we had stored samples for 77 study subjects who completed the one year of treatment (38 in the placebo group and 39 in the statin group). Accordingly, data are presented as a per-protocol analysis. Serial changes were studied using analysis of variance (ANOVA) for repeated measurements (RM-ANOVA), with treatment group as the independent variable (effect of time by group). Assumptions for repeated measures ANOVA were checked by Mauchly's test of sphericity, and accordingly adjustment in the degrees of freedom was made (Huynh-Feldt epsilon). In case of a significant between-groups difference by repeated measures ANOVA, differences between groups were analysed at each time point of measurements by a posterior analysis using a two-sample test. We assessed association between studied parameters using correlations and multiple regression analyses. All results are given as mean ± standard error of the mean (SEM) unless otherwise stated. Statistical analysis was performed using Statistical Package for Social Sciences (SPSSs 14.0) for Windows.

## 5. Results

Women included in the study had a median age of 64 years (range 53 to 74 years). Baseline characteristics including P-25OHD levels did not differ statistically between groups, except that women randomised to placebo by change were heavier than subjects randomised to simvastatin treatment (Table 1). Only three of our studied subjects had vitamin D deficiency defined as a P-25OHD level <25 nmol/L, whereas 11 (14%) had vitamin D insufficiency (P-25OHD <50 nmol/L). One third of our studied subjects had a P-25OHD level >80 nmol/L (Table 1). As previously reported, 52 weeks of treatment with simvastatin caused, compared with placebo, a significant decrease in plasma levels of cholesterol (−27%, 95% confidence interval (CI), −22% to −32%, *P* < .001) and LDL-cholesterol (−45%, 95% CI, −38% to −52%, *P* < .001), whereas plasma levels of HDL-cholesterol and triglycerides did not change significantly in response to treatment [1]. Similarly, statin treatment did not affect plasma levels of parathyroid hormone or biochemical markers of bone turnover, including plasma levels of C-terminal telopeptide of type I (CTX), collagen osteocalcin, and bone specific alkaline phosphatase [1].

### 5.1. Effects of Simvastatin Treatment on Plasma 25OHD Levels

**Figure 1:**
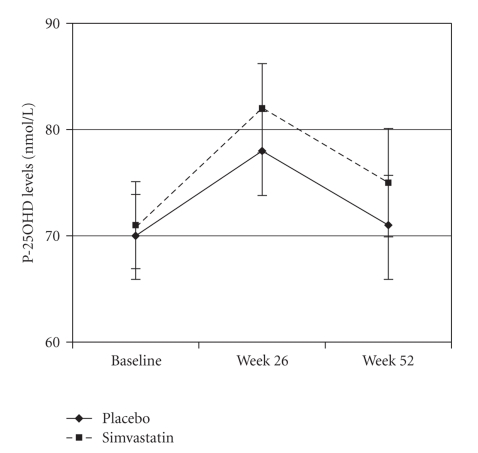
Changes in plasma 25-hydroxyvitamin D (P-25OHD) levels by treatment group (mean ± SEM).

Treatment with simvastatin did not affect P-25OHD levels compared with placebo (*P* = .53 by RM-ANOVA, Figure 1). P-25OHD levels did not change significantly between baseline and week 52 within the group of women treated with simvastatin (*P* = .40 by paired sample test) or placebo (*P* = .90). As shown in Figure 1, P-25OHD levels were higher in both study groups at week 26 compared with values at baseline and week 52, which is attributable to seasonal variations. Samples at baseline and week 52 were collected during wintertime (October to April), whereas samples at week 26 were collected during summertime (April to October).

### 5.2. Associations between Vitamin D- and Cholesterol-Status

In order to assess whether P-25OHD levels influence cholesterol status, we analysed baseline indices of cholesterol status, as measured by plasma levels of TC, LDL, HDL, or TG by tertiles of P-25OHD levels (Table 2). Women in the highest tertile of P-25OHD levels (>80 nmol/L) had significantly (*P* < .01) lower plasma levels of TG than those in the lowest P-25OHD tertile, whereas plasma HDL levels increased borderline significantly by P-25OHD tertiles (Table 2). Dividing studied subjects into groups pf vitamin D status according to the often used cutoff limits for vitamin D status, that is, P-25OHD <50, between 50–80, and >80 nmol/L showed very similar results with significantly lower TG levels (1.0 ± 0.5 mmol/L) in vitamin D replete women (P-25OHD >80 nmol/L) than in women with vitamin D insufficiency (P-25OHD <50 nmol/L: TG 1.3 ± 0.5 mmol/L, *P* = .03). Moreover, on averages, P-25OHD levels increased from 71 ± 25 nmol/L at wintertime (baseline) to 80 ± 25 nmol/L at summertime (week 26). Concomitantly, plasma TG levels decreased from 1.2 ± 0.5 mmol/L to 1.1 ± 0.5 mmol/L. In a linear regression analysis, adjusted for treatment allocation, the seasonal changes in P-25OHD levels were significantly associated with the concomitant changes in plasma TG levels (*β* = −0.150, *r* = 0.47, *P* < .01). Similarly, the decrease in P-25OHD levels between week 26 and 52 (from summer- to winter-time) correlated significantly with the concomitant changes in plasma TG levels (*β* = −0.189, *r* = 0.36, *P* < .01). Further adjustments for BMI did not change the results.

## 6. Discussion

In a randomised, controlled study, we found no effects on plasma 25OHD levels of one year of treatment with simvastatin 40 mg/d compared with placebo. However, our analysis showed an effect of vitamin D status on plasma levels of TG, a finding that may contribute to our understanding of the potential positive effects of vitamin D on cardiovascular health.

For more than two decades, statins have been used to reduce cholesterol levels in patients with cardiovascular diseases. They act as HMG-CoA reductase inhibitors, thereby reducing the endogenous cholesterol synthesis. When statins were introduced, it was a matter of concern whether inhibition of the cholesterol biosynthetic pathway may affect other metabolic processes which are dependent on intermediates from this pathway. Especially, concerns have been paid to the reduced tissue concentrations of 7-dehydrocholesterol (7-DHC) in response to treatment with statins. As 7-DHC is the precursor for endogenous skin synthesis of cholecalciferol, reduced levels of 7-DHC may impair vitamin D status. However, in a study including 17 men and women on treatment with pravastatin and 14 hypercholesterolemic age and gender matched controls, vitamin D levels increased in a similar manner in both groups in response to exposure of the skin surface to type B ultraviolet (UV-B) radiation, indicating no harmful effects of pravastatin on the endogenous vitamin D synthesis [19]. On the contrary, in several papers statin therapy has been suggested to improve vitamin D status. In a group of 83 Spanish men and women with acute coronary syndrome in whom treatment with atorvastatin was initiated, vitamin D status as measured by P-25OHD levels improved. Thus, during one year of observation P-25OHD levels increased from 41 ± 19 nmol/L at baseline to 47 ± 19 nmol/L after 12 months, which, according to the investigators, was attributable to treatment with atorvastatin [24]. Similar results have been reported by other investigators [15, 16], including a cross-sectional analysis showing increased P-25OHD levels in patients on treatment with statins [14]. In contrast to these findings from observational studies, the results from our randomised controlled study showed no effect of simvastatin treatment on P-25OHD levels. Although we cannot exclude differential effects on vitamin D status of different types of statin drugs (e.g., atorvastatin versus simvastatin), we find it most likely that the findings from the uncontrolled studies are due to unmeasured changes in indices affecting P-25OHD levels. In general, P-25OHD levels are determined by intake from food items rich in vitamin D (especially fatty fish), use of vitamin D supplements, and sun exposure. To the best of our knowledge, none of these indices was controlled for in the hitherto published uncontrolled studies on possible effects of statins on vitamin D status. Most likely, the findings from these studies are due to the coincidence of changes in life-style habits in relation to administration of statin drugs.

Vitamin D has, however, independently of treatment with statins, been implicated in cardiovascular health and atherogenesis [25]. Results from a randomised controlled study showed a decreased blood pressure in response to UVB exposure [26], and P-25OHD levels have been shown to correlate inversely with risk of atherosclerosis in several cohort studies [21, 27, 28]. However, other investigators have reported either no effects or even detrimental effects on plasma lipid levels in response to administration of vitamin D [29–32]. The vitamin D receptor is expressed by both cardiac myocytes, endothelial-, and smooth vascular muscle-cells and these cells also express the 1 *α*-hydroxylase enzyme, that is, they may locally activate vitamin D from its circulation precursor (25OHD) to its active metabolite (1,25-dihydroxyvitamin D) [33, 34]. Several mechanisms of action have been implicated in the potential antiatherosclerotic effects of vitamin D, including a downregulation of the renin-angiotensin system, and a reduced expression of mRNA and protein levels of plasminogen activator inhibitor-1 (PAI-1) and thrombospondin-1 (THBS1) which are known to be involved in the development of atherosclerosis [35, 36]. In addition, our data points toward a further mechanism by which vitamin D may protect against cardiovascular diseases, that is, through decreased TG levels [37]. A possible mechanism of action by which vitamin D lowers TG levels is through an increased activity of the lipoprotein lipase, which has been shown to be regulated by vitamin D in adipocytes [38]. Our data on effects of vitamin D on TG levels are limited by the cross-sectional design of the analysis and included only women. Further randomised controlled studies on effects of vitamin D on plasma lipid profile are warranted, including studies in men.

In conclusion, results from our randomised, controlled trial do not support an effect of statin treatment on vitamin D status as determined by measurement of plasma 25OHD levels. However, P-25OHD levels may improve the plasma lipid profile and thereby risk of cardiovascular disease.

## Figures and Tables

**Table 1:** Baseline characteristics by study group (mean ± sem).

	Placebo (*n* = 38)	Simvastatin (*n* = 39)	*P*-value (*n* = 77)
Age (years)^(1)^	64 (53–72)	63 (53–72)	.70
Years postmenopausal^(1)^	18 (6–44)	19 (7–31)	.80
Scale body weight (kg)	69.6 ± 1.4	64.6 ± 1.8	.04
Body mass index (kg/m^2^)	26.4 ± 0.5	24.7 ± 0.7	.04

Biochemestry			
*Plasma *			
Calcium (adj.) (mmol/L)	2.44 ± 0.01	2.46 ± 0.01	.17
Creatinine (mol/L)	77 ± 2	78 ± 2	.82
Total cholesterol (mmol/L)	6.4 ± 0.1	6.5 ± 0.2	.69
HDL cholesterol (mmol/L)	1.8 ± 0.1	2.0 ± 0.1	.14
LDL cholesterol (mmol/L)	4.0 ± 0.1	4.0 ± 0.2	.99
Triglycerides (mmol/L)	1.3 ± 0.1	1.1 ± 0.1	.08
P-PTH (pmol/L)	4.2 ± 0.2	4.4 ± 0.2	.39
25OHD (nmol/L)	70 ± 4	71 ± 4	.89
25OHD <25 nmol/L, *N* (%)	2 (5%)	1 (3%)	.54
25OHD <50 nmol/L, *N* (%)	7 (18%)	4 (10%)	.35
25OHD <80 nmol/L, *N* (%)	25 (66%)	26 (67%)	.94

^(1)^Median (range).

**Table 2:** Indices of cholesterol status by tertiles of plasma 25-hydroxyvitamin D (P-25OHD) levels at baseline (mean ± SD).

	P-total cholesterol	P-HDL cholesterol	P-LDL cholesterol	P-triglycerides
	(mmol/L)	(mmol/L)	(mmol/L)	(mmol/L)
Tertile of P-25OHD levels				
(1) (<58 nmol/L)	6.4 ± 1.1	1.7 ± 0.4	4.4 ± 1.0	1.3 ± 0.6
(2) (58–80 nmol/L)	6.6 ± 0.8	2.0 ± 0.6	4.1 ± 0.8	1.3 ± 0.5
(3) (>80 nmol/L)	6.6 ± 1.1	2.0 ± 0.5*	4.1 ± 1.0	1.0 ± 0.5*
p-trend	0.52	0.06	0.98	<0.01

*P < .05 compared with lowest tertile by post-hoc test.
